# Volatolomics of Sardinian and Spanish *Bituminaria*: Characterization of Different Accessions Using Chemometrics

**DOI:** 10.3390/molecules26175247

**Published:** 2021-08-30

**Authors:** Marianna Usai, Mauro Marchetti, Rita A.M. Melis, Claudio Porqueddu

**Affiliations:** 1 Department of Chemistry and Pharmacy, University of Sassari, via Muroni 23/a, 07100 Sassari, Italy; 2 Institute of Biomolecular Chemistry, National Research Council (CNR), Trav. La Crucca 3, 07100 Sassari, Italy; mauro.marchetti@cnr.it; 3 Institute for the Animal Production System in the Mediterranean Environment (CNR), Traversa La Crucca 3, 07040 Sassari, Italy; rita.melis@cspm.ss.cnr.it (R.A.M.M.); claudio.porqueddu@cspm.ss.cnr.it (C.P.)

**Keywords:** *Bituminaria genus*, *Bituminaria morisiana*, essential oils, chemometrics tool, furocoumarins

## Abstract

The present study aims to determine the volatile compositions of 15 different accessions of native Sardinian populations of *Bituminaria morisiana* (Pignatti & Metlesics) Greuter, *Bituminaria bituminosa* (L.) C. H. Stirt. (*B. b.*), and Spanish native accessions of *B. bituminosa*. Furthermore, we particularly focused on the essential oil characterization of these accessions and discriminated within populations with low furocoumarin content useful for fodder production in Mediterranean environments or furocoumarin extraction for pharmaceutical utilization. The plant extracts were analyzed by GC/MS, showing great variability in the content and composition. No differences were found in *Bituminaria bituminosa* (L.) C.H. Stirt. var. *bituminosa* essential oils, while the varieties *Bituminaria bituminosa* (L.) C.H. Stirt. var. *crassiuscula* P. Méndez, Fern. Galván & A. Santos and *Bituminaria bituminosa* (L.) C.H. Stirt. var. *albomarginata* P. Méndez, Fern. Galván & A. Santos are characterized by the presence of a high concentration of long-chain alcohols and of salicylic acid benzylic ester. In *B. bituminosa* var. *albomarginata*, we observed a different profile with predominance of a large concentration of alcohols as dodecanol and tetradecanol. The endemic *B. morisiana* can be identified for the predominant presence of farnesene. In methanolic fractions, we detected the presence of maltol, methyl citrate, methyl cumarate, santonin, and methyl linoleate. *B. morisiana* showed a low content of psoralens, and the accession of *B. morisiana,* from Siliqua indicated the presence of apocynin.

## 1. Introduction

*Bituminaria* genus (Fabaceae) was first established by Linnaeus in the second edition of the Linnaeus Gen. Plant., 358 (I742) and credited to Royen. In the first edition of the Sp. Plant., 762 (1753), eight species are described, only one of which is credited to America, and it is probably an introduced plant from the Island of Madeira [1].

The morphology of the genus *Bituminaria* Heist. ex Fabricius (Psoraleeae, Fabaceae) is very heterogeneous and it is widespread across the Mediterranean region and Macaronesian Islands, where it is estimated to have diverged from other Psoraleoid genera approximately 6.78 million years ago. The causes of diversification can be attributed to various factors, such as habitat modifications and reproductive biology. These speciation processes on the *Bituminaria* genus led to eight distinct species: *B. bituminosa* (L.) C.H. Stirt., *B. morisiana* (Pignatti & Metlesics) Greuter, *B. flaccida* (Nábělek) Greuter, *B. basaltica* Miniss., C. Brullo, Brullo, Giusso & Sciandr., *B. kyreniae* Giusso, C. Brullo, Brullo, Cambria & Miniss., *B. palaestina* (Bassi) Brullo, C. Brullo, Miniss., Salmeri & Giusso, *B. plumosa* (Rchb.) Bogdanović, C. Brullo, Brullo, Ljubičić & Giusso, and *B. acaulis* (Steven ex M. Bieb.) C.H. Stirt. [2].

In Europe, only *Bituminaria bituminosa* (L.) C.H. Stirt. var. *bituminosa* is present, which is a perennial legume from the Mediterranean Region and is, therefore, also able to resist drought and a hot climate. *Bituminaria* grows in the Mediterranean basin and Macaronesia, and it has a potential as pasture fodder for ruminants in semiarid environments, but only few selected ecotypes are cultivated in Canary Islands and Morocco for this purpose [3].

On Sardinia Island, two species of *Bituminaria* can be found in natural stands: *B. bituminosa* var. *bituminosa* and the endemic *Bituminaria* *morisiana* (Pignatti & Metlesics) Greuter. They can be easily distinguished on the basis of some morphological traits and the presence of a typical bitumen smell in *B. bituminosa* [4]. In Spain and the Canary Islands, *B. bituminosa* is spontaneously grown, which exhibits a large diversity with three botanical varieties *Bituminaria bituminosa* var. *albomarginata* (albo tedera), *Bituminaria bituminosa* var. *crassiuscula* (teide tedera), and *Bituminaria bituminosa* var. *bituminosa* (tedera). This genus also grows across the Mediterranean basin, and it is known as Arabian pea or pitch trefoil [3,5,6]. Both species have an appreciable content of furocoumarins, compounds that are related to a low palatability of fresh fodder [7], but that can be used in therapy combining the oral drug psoralen and high-intensity longwave ultraviolet light (PUVA) [8].

Only few studies have been carried out on the furocoumarin content of *Psoralea* genus and *Bituminaria* genus, mostly on *Psoralea corylifolia* L. [9,10,11,12]. On *B. bituminosa* and its varieties, the literature is very scarce [13,14,15,16,17].

As far as the knowledge on the composition of the essential oil of *Bituminaria* genus is concerned, the paper of Bandeira et al. described the chemical composition of essential oil of *Bituminaria basaltica* Miniss., C. Brullo, Brullo, Giusso & Sciandr. [17], whereas other authors have investigated essential oils in *Psoralea drupacea* Bunge and *P. corylifolia* L. In these species, the presence of bakuchiol, angelicin, α-pinene, and limonene [18] and their antimicrobial activities [19,20] were documented. Volatile composition was also assessed in different organs of *B. bituminosa* collected in Tuscany [21]. Another study on a *B. bituminosa* germplasm collected in Italy was performed on the volatile composition of fresh leaves and flowers collected at the experimental field of the University of Tuscia, Viterbo [22]. El-Seedi et al. investigated the essential oil of *Psoralea pubescence* (Miq.) Standl in plants collected at the Reserva Forestal ENDESA (Ecuador) [23]. A study on *B. bituminosa* var. *bituminosa* essential oil growing wild in Elba Island was published in 2016 [14].

*Bituminaria* genus is widely present in Sardinia, along with the endemic *B. morisiana,* which is very interesting for its metabolites. Various articles on *B. morisiana* have revealed the presence of pterocarpans [24]; in particular, a paper on *B. morisiana* characterized two new pterocarpans and demonstrated a low cytotoxic activity of erybraedin C on all used cell lines, while erybraedin C induced necrosis in leukemia Jukart T cells [25].

Recently, 15 accessions of *Bituminaria*: *B. morisiana* (Sardinian endemism), *B. bituminosa*, and *B. bituminosa* var. *albomarginata*, *crassiuscula* and *bituminosa*, of Sardinian and Spanish origin, were studied for their forage yield, furocoumarins, and essential oil content [26,27]. Furocoumarin extracts from *B. morisiana* were also tested for their mutagenic potential on river buffalo blood cells [28].

In this view, we conducted a comparative analysis among native Sardinian populations of *B. morisiana* and *B. bituminosa* and Spanish native accessions of *B. bituminosa* and some of its varieties. The analyses of EOs of all studied plant species were examined using chemometric tools (PCA). The aim was to characterize the volatile compounds discriminating the population of these accessions to be used in the health or herbal field. In this way, it we had the possibility to divide accessions with low furocoumarin content to use for fodder production in Mediterranean environments and accessions with high furocoumarin content useful as pharmaceutical preparations.

## 2. Results

### 2.1. Chemical Composition of the Essential Oil Content

We analyzed 15 different accessions of plants belonging to the *Bituminaria* genus and growing in experimental fields of the Interdepartmental Center for the Conservation and Enhancement of Plant Biodiversity of Sassari University, coming from different areas of Sardinia and Spain. From the aerial part of fresh plants, we extracted and characterized the essential oils and the furocoumarins.

All the obtained essential oils were diluted at the same concentration and subjected to GC and GC/MS characterization; considering all the species, 96 constituents were identified.

In the six *B. bituminosa* var. *bituminosa* samples, three collected in Sardinia and three collected in Spain, the identified components were never lower than 91.45% (Table 1). 

In the essential oil obtained from the accessions of *B. bituminosa* var. *bituminosa* coming from the Sardinian populations, we identified 63 different compounds, which accounted for 98.39%, 97.30%, and 94.34% of constituents.

In the three populations native to Sardinia, among all constituents, 22 had a concentration higher than 1%. In Loculi, the major constituent was caryophyllene oxide (19.56%). β-Caryophyllene was the major constituent of Sassari station (17.30%). Whereas germacrene D was the more important constituent in the accession coming from Siniscola (10.46%), it had a minimum level in Loculi (5.38%).

An interesting aspect concerning the composition of these essential oils was the presence of long-chain alcohols such as caryophylla-4(12),8(13)-dien-5α-ol, which was present only in concentrations lower than 1%, caryophylla-4(14),8(15)-dien-5β-ol, found in three stations with concentrations ranging between 2.30% and 1.44%, (*cis*,*cis*)-9,12-octadecadien-1-ol, present in good concentration (5.28%) in Sassari and in Loculi (3.87%), reaching the minimum concentration (1.85%) in Siniscola, and (*cis*,*cis*,*cis*)-9,12,15-octadecatrien-1-ol, reaching a very similar concentration at all Sardinian stations (4–5%). Another aspect was the high concentration of oxides.

In *B. bituminosa* var. *bituminosa*, derived from Spanish seeds or seedlings (58 identified constituents), the main constituents differed in each sample. In LIano del Beal, the main constituent was β-caryophyllene (34.47%); the other well-represented components were *cis*-β-farnesene (8.19%) and α-humulene (7.75%). In Calnegre samples, the main component was *cis*,*cis*-9,12-octadecadien-1-ol (15.76%). In San Cristòbal de la laguna samples, we found a different distribution of principal constituents; the main components were (*cis*,*cis*)-9,12-octadecadien-1-ol (11.06%), β-caryophyllene (8.18%), (*cis*,*cis*,*cis*)-9,12,15-octadecatrien-1-ol (6.58%), and caryophyllene oxide (5.58%).

In samples from accessions of *B. bituminosa* var. *albomarginata* (Canary Islands) (Table 2), we identified 47 constituents representing about 93.8% of the total and a different composition between the two stations was found.

In *B. b. albomarginata* accessions coming from Caleta de Famara, the main constituents were long-chain alcohols (*cis*,*cis*)-9,12-octadecadien-1-ol (14.96%) and (*cis*,*cis*,*cis*)-9,12,15-octadecatrien-1-ol (9.86%). In *B. b. albomarginata* accessions coming from Arecife, an original profile was found. In fact, there was a predominance of lauryl alcohol (13.83%) (not present in Caleta de Famara) and tetradecanol (10.93%).

*B. bituminosa* var. *crassiuscula* belonging from Spanish native plants was characterized by the presence of 49 constituents, representing about 97% and 94% of the total. Among these constituents, 21 exceeded 1%. A common feature of oils at these two stations was represented by a high concentration of β-caryophyllene (22–25%) and caryophyllene oxide 11% in Vilaflor and about 7% in San Cristòbal de la laguna. A component present only in these two oils was benzyl salicylate, which is a compound frequently used in cosmetics as a fragrance or UV light absorber [42]. Another constituent present in high concentration in these varieties was benzyl benzoate (3.05% and 2.38%), which is also used in cosmetics as fragrance but is better known for the topical treatment of human scabies [43].

In *Bituminaria morisiana* Pign. et Metlesics (Sardinian endemism), it was possible to identify 54 constituents of the essence in toto, representing from 93.41% to 96.75% of the total (Table 3). 

Twenty-six constituents reach a concentration higher than 1%. Five were present in a considerable amount. *cis*-β-Farnesene was the main constituent in all samples, from a minimum of 26.91% in Bitti accession to a maximum of 41.77% in Punta Giglio accession. The concentration in the other three stations varied from 34.36% to 37%. Other constituents present in very high concentration were two long-chain alcohols (*cis*,*cis*)-9,12-octadecadien-1-ol (linoleyl alcohol) from a minimum of 9.72% in Bitti to a maximum of 17.73% in Punta Giglio. Germacrene D was present in all samples with a high concentration in Monte Gonareddu and a minimum in Punta Giglio 2.84%. β-Caryophyllene was well represented in the two stations of Bitti and Siliqua with concentrations of 9.92% and 9%, respectively. In Bitti accession, 21.76% of alcohols deriving from fatty acids were present, as well as in Punta Giglio (22.32%), while, in the other stations, fatty alcohols reached a concentration range between 16% and 18%. 

### 2.2. Methanolic Extracts

**Figure 1:**
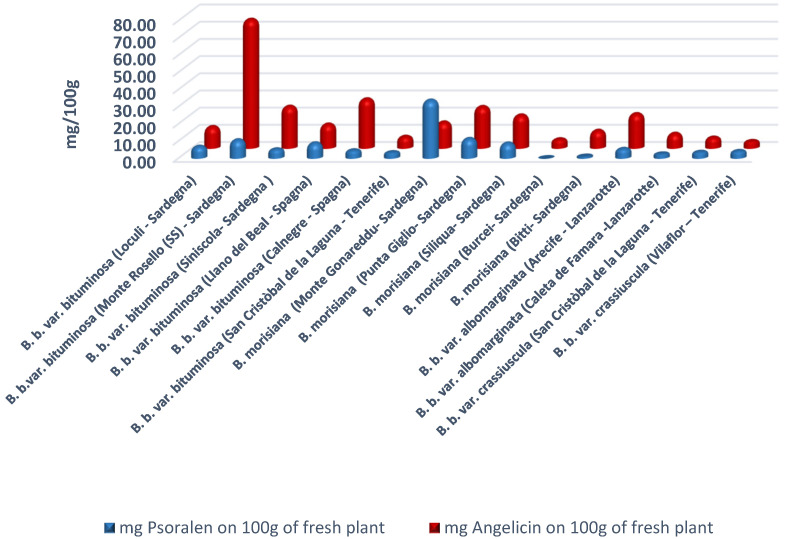
Content of psoralen and angelicin in the analyzed *Bituminaria* species and varieties (mg/100 g, fresh plant).

The methanolic extracts were partitioned using H_2_O/CHCl_3_ and analyzed by GC/MS (see Section 4) [44]. From the methanolic extracts, we obtained 15 samples that were analyzed by GC and GC/MS. The content of furocoumarins is expressed in mg/100 g of fresh plant (Figure 1). In the six analyzed samples of *B. b.* var. *bituminosa,* Monte Rosello showed a very high content of furocoumarins. In these samples, we found 750.2 mg/kg angelicin and 108.7 mg/kg psoralen calculated in fresh plant. In general, all samples from *B. b.* var. *bituminosa* showed a larger quantity of angelicin than psoralen. The analyzed extracts from *B. b.* var. *bituminosa* species from plants coming from Sardinia and Spain showed, in addition to the expected furocoumarin content, good amounts of maltol, trimethyl citrate, methyl coumarate, santonin, and methyl linoleate. In the crude extracts derived from our samples, we found a good concentration of maltol (e.g., 34.71% in Calnegre and 31.56% in San Cristòbal de la Laguna). In the Sardinian samples, the maximum content of maltol in the crude extract was found in the accession derived from Loculi (14.89%). In the Spanish varieties, salicylic acid was found in the crude methanol extract, varying from 2.78% in San Cristòbal de la laguna *B. b.* var. *bituminosa* to 0.35% in LIano del Beal *B. b.* var. *bituminosa*. The other varieties from Spain showed the highest concentration of maltol in *B. b.* var. *crassiuscula* from Vilaflor (39.32%), while a high concentration (33.96%) was also found in San Cristòbal de la laguna station.

The endemic species of Sardinia, *B. morisiana*, generally showed a low content of furocoumarins, while the accessions native to Burcei did not contain more than 55 mg/kg of these substances. An exception is constituted by *B. morisiana* derived from the accession of Monte Gonareddu, which surprisingly had a high content of furocoumarins (psoralen 339.4 mg/kg and angelicin 154.3 mg/kg). 

In terms of the other characterized compounds, in the endemic *B. morisiana*, maltol reached a maximum of 19.63% in the crude extract derived from the Siliqua station accession. Only in the extract derived from *B. morisiana* from Siliqua was a small quantity of apocynin found (1.54%).

### 2.3. Statistical Analysis

**Figure 2:**
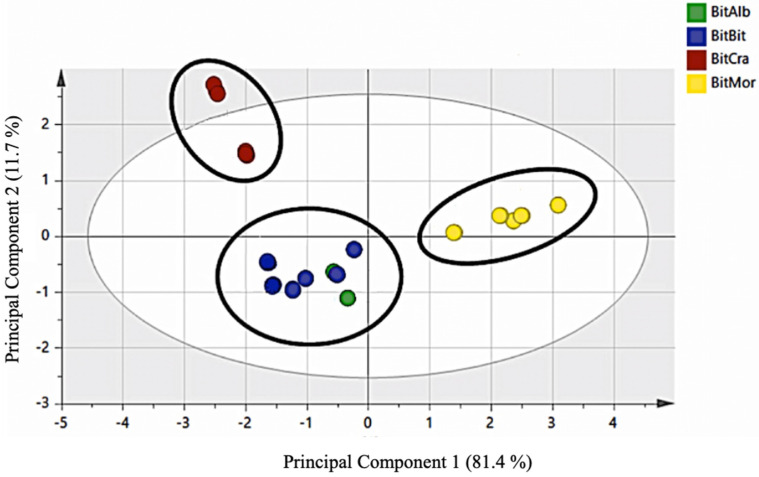
Score plot of PCA analysis performed using the essential oil components. Principal components 1 and 2 represented 93.1% of the variance.

The obtained data were submitted to multivariate statistical evaluation to verify the biodiversity through secondary metabolites. The PCA analysis performed using only essential oil components (96 different compounds were identified) (Figure 2) clearly shows three different groups of plants; *B. b.* var. *bituminosa* and *B. b.* var. *albomarginata* were grouped, showing the homogeneity of secondary metabolites present in the essential oil. On the contrary, the accessions of *B. b.* var. *crassiuscula* and *B. morisiana* were well-defined groups.

**Figure 3:**
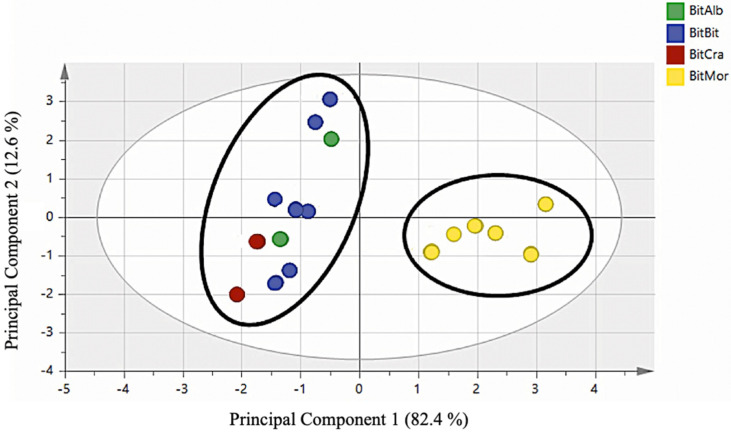
Score plot of PCA statistical analysis of *B. morisiana* and *B. bituminosa* varieties. Principal components 1 and 2 represented 95.0% of the variance.

Using all identified volatile metabolites coming from water distillation and methanolic extraction for PCA statistical analysis, it was possible to distinguish *B. b.* var. *morisiana* from *B. bituminosa* and all its varieties. Figure 3 reports the plot score of this analysis.

## 3. Discussion

The plants we analyzed showed great variability with regard to the content and the composition of essential oils. It is not easy to identify the accessions of *B. b.* var*. bituminosa* because there are no decisively discriminating constituents. Comparing the samples coming from Sardinian accessions and that from native plants from Spain, we can note that the most represented constituents were always the same, albeit in different concentrations. A twofold higher concentration of β-caryophyllene was found in LIano del Beal compared with Sardinian samples. In other compounds, e.g., caryophyllene oxide, there were no significant differences in concentration.

Comparing *B. b.* var. *albomarginata* and *B. b.* var. *crassiuscula*, it was possible to note some differences; in *B. b.* var. *albomarginata* with respect to *B. b.* var. *crassiuscula*, we found a higher concentration of α-copaene, germacrene D, and palmitic acid. Moreover, in *B. b.* var. *albomarginata*, some compounds were present in contrast to *B. b.* var. *crassiuscula*, such as lauryl alcohol, β-sesquiphellandrene, *n*-tetradecanol, and viridiflorene. Benzyl benzoate, also present in some other varieties, reached its maximum in *B. b.* var. *crassiuscula*. *B. b.* var. *crassiuscula* also contained long-chain alcohols and benzyl salicylate.

In *B. morisiana*, we observed the preponderant presence of farnesene, unique to this essential oil; moreover, germacrene D was present in all samples with a high concentration in Monte Gonareddu and a minimum concentration in Punta Giglio. This volatile sesquiterpene belonging to the germacrene family is typically produced in plants as an antimicrobial and insecticidal or as an insect pheromone.

The concentration of essential oils distilled from *Bituminaria* genus was to predict their use in pharmaceutical applications, especially in terms of furanocoumarins such as psoralen and angelicin. Psoralens are widely used to treat human skin diseases and for their antimicrobial activity and anti-HIV effects. Angelicin has calmative, sedative, and anticonvulsant activities and is used for the treatment of thalassemia (US Patent No.: US2006/0111433(A1)). On this basis, we proceeded to quantify the concentration of furocoumarins and other volatile products, which are easily extracted by methanol, such as maltol, trimethyl citrate, methyl coumarate, santonin, and methyl linoleate, which might have medicinal interest. All these substances give to these plants an interest that goes beyond the simple furocoumarin content and enhance their effect. For instance, maltol itself is known for its odor of cotton candy and caramel and is used to impart a sweet aroma to fragrances. Some synthetic derivatives of maltol showed limited in vitro antiproliferative activity toward cancer cells lines [45].

Analyzing all the varieties, we found many other compounds having pharmaceutic interest. For example, in all Spanish varieties, we found salicylic acid, which is an anti-inflammatory that might increase the effect of furocoumarins. In the extract derived from *B. morisiana* from Siliqua, we found a small quantity of apocynin, which is a very interesting compound, since it is a selective inhibitor of the phagocyte NADPH oxidase Nox2, which can be applied orally and is remarkably effective at low dose [46].

The quantified furocoumarins in our samples (Figure 1) were very significant, indicating a strong correlation between the unpleasant smell of the plant and the content of psoralens. In particular, the accessions native to Burcei did not contain more than 55 mg/kg of these substances, and it is very interesting to note that these species did not exhibit the characteristic odor of bitumen. On the contrary, other accessions contained a relatively large amount of furocoumarins; these plants could be really exploited in phytotherapy as a furocoumarin source, e.g., in the treatment of psoriasis according to the BALNEO-PUVA methodology (BATH-PUVA) [47]. In this view, the fraction extracted with the methanol/acidic water solution partitioned in H_2_O/CHCl_3_ (see Section 4) proved to be a valuable source of anti-inflammatory compounds and may be well suited for the treatment of psoriasis using the BALNEO-PUVA methodology. In particular, the accession of *B. b.* var. *bituminosa* from Monte Rosello would be able to easily provide the concentration of furocoumarin necessary for this health treatment.

*B. b.* var. *albomarginata* and *B. b.* var. *crassiuscula* derived from Spain accessions showed a low content of psoralens. 

The obtained data coming from essential oil analyses or methanol extract analyses were submitted to multivariate statistical evaluation to verify the biodiversity through secondary metabolites and furnished a very interesting indication, enabling the distinction of *B. morisiana* from *B. bituminosa* and its varieties (Figure 3). Moreover, using only the essential oil components, it was possible to divide the accessions into different groups of plants. *B. b.* var. *bituminosa* and *B. b.* var. *albomarginata* were grouped together, showing the homogeneity of secondary metabolites present in their essential oils. On the contrary, the accessions *B. b.* var. *crassiuscula* and *B. morisiana* were well defined. These results are interesting if compared with those published by A. Muñoz et al. [3], where morphological and molecular analyses were used in the principal component analysis and enabled the characterization of different *B. bituminosa* accessions coming from southeast Spain and the Canary Islands into six homogenous groups. The authors reported that *B. b. var. crassiuscula* can be well differentiated from other *Bituminaria* plants in terms of its morphological characteristics.

## 4. Materials and Methods

### 4.1. Chemicals and Instrumentation

The fertilization of plant material was carried out using P_2_O_5_ (NIT Greenhouse grade 25 kg) from Haifa-Italia srl (Bologna, Italy). The distilled water used for the essential oil isolation was produced in the laboratory, whereas anhydrous Na_2_SO_4_ was obtained from Merck S.p.A. (Sigma-Aldrich) (Milano, Italy). The quantitative GC analyses were performed using a Hewlett-Packard Model 5890A GC equipped with an automatic injector HP 7673 (now Agilent Technologies Italia S.p.A, Milano, Italy) and GC/MS analyses were conducted using an Agilent Technologies model 7820A Milano, Italy connected with a MS detector 5977E MSD (Agilent Technologies Italia S.p.A, Milano, Italy) equipped with an automatic injector HP 7673 for qualitative analyses. The carrier gas helium was obtained from SAPIO, and the columns (Phenomenex ZB-5, Torrance, CA, USA) for GC and GC/MS analyses were from ThermoFisher scientific Italia (Monza, Italy). The 400 MHz NMR spectra were recorded using a VARIAN (Mercury plus) spectrometer (now Agilent Technologies Italia S.p.A, Milano, Italy).

### 4.2. Plant Material 

**Figure 4:**
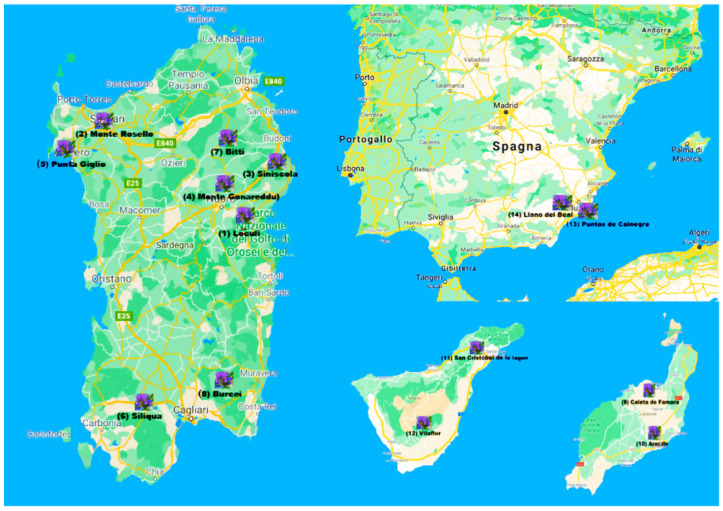
Collecting stations for accessions of Sardinian and Spanish *Bituminaria* plants.

An experimental field was established in CBV (Interdepartmental Center for the Conservation and Enhancement of Plant Biodiversity Center) of Sassari University located in Surigheddu district (40°35′49″ N; 8°22′47″ E) close to Alghero in northwest Sardinia. The site has a Mediterranean climate with an average annual rainfall of 540 mm and an alluvial soil with a high limestone content and neutral pH (6.9). The cultivation of different species and varieties of *B. bituminosa* was carried out by researchers of ISPAAM Institute (Institute for the Animal Production System in the Mediterranean Environment) of CNR (Research National Council), who handled the germplasm. They oversaw the bio-morphological screening concerning the accessions collected from different stations of Sardinia, and they took care of all agronomic factors, seed production, and its components. They also planted the seeds from the Spanish accessions. The samples of plant material derived from certified seeds or plantlets were treated after collection to avoid the degradation of biomass. The collecting stations for Sardinian *B. b.* var. *bituminosa* plants are reported in Table 4 and shown in Figure 4.

The species located in Canary Islands showed a large diversity, with three botanical varieties found in habitats ranging from the coastal semiarid areas on Lanzarote Island with an annual rainfall of 150–300 mm (*B. b.* var. *albomarginata*) to the high elevation subhumid area (1700–2200 m, 500 mm) of Tenerife (*B. b.* var. *crassiuscula*). The third (*B. b.* var. *bituminosa*) displayed a wide adaptation across the Canary Islands (300–1000 m) and is the only one present in the Mediterranean basin. In the Iberian Peninsula, it has been found in environments ranging from 250–1000 mm of rainfall and up to 1250–1500 m of altitude [48]. Germplasms of *Bituminaria* species and varieties were collected in different Spanish areas (Table 4). The experiments were carried out in plots with 12 plants per accession in a completely randomized design with three replicates. Plants were grown from scarified seeds sown in jiffy pots in a greenhouse and then transplanted to the field in February. Fertilization was done before planting with 46 kg·ha^−1^ of P_2_O_5_. Occasional irrigation was supplied to plants, when necessary, from late spring to early summer in the first year.

### 4.3. Essential Oil Extraction

**Figure 5:**
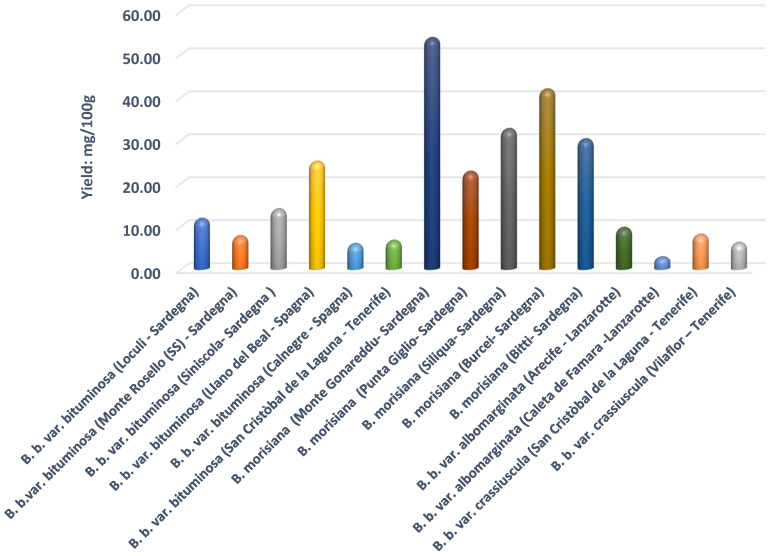
Essential oil yield (mg/100 g) of *Bituminaria* species and varieties.

Plant material (200 g of aerial part) of 15 accessions of *Bituminaria* genus was submitted to hydrodistillation for 4 h using a Clevenger-type apparatus. The oils were collected by separation from the aqueous phase, dried over anhydrous Na_2_SO_4_, and stored at −20 °C before being analyzed. The reached yields (*w*/*w*) are reported in Figure 5. 

### 4.4. Gas Chromatography and Mass Spectrometry (GC/MS) Analysis

Three replicates of the essential oils were separately analyzed using a GC (Hewlett-Packard Model 5890A, Agilent Technologies Italia S.p.A, Milano, Italy) equipped with a flame ionization detector and fitted with a 60 m × 0.25 mm, thickness 0.25 μm ZB-5 fused silica capillary column (Phenomenex). The injection port and detector temperatures were 280 °C. The column temperature was programmed from 50 °C to 135 °C at 5 °C/min (1 min), 5 °C/min up 225 °C (5 min), 5 °C/min up 260 °C, and held for 10 min. Oil samples of 0.2 μL injection volume were analyzed, diluted in hexane using 2,6-dimethylphenol as an internal standard. Injection was performed using a split/splitless (used in split mode, ratio 50:1) automatic injector HP 7673 and helium as a carrier gas. Several measurements of peak areas were performed through an HP workstation with a threshold set to 0.00 and peak width set to 0.02. Compound quantification was expressed as absolute weight percentage using internal standard (*n*-tetradecane) response factors (RFs). Since oxygenated compounds have lower detectability than hydrocarbons by FID, detector RFs were determined for key components relative to 2,6-dimethylphenol and assigned to other components on the basis of functional group and/or structural similarity. MS analyses were carried out with an Agilent Technologies model 7820A connected to an MS detector 5977E MSD (Agilent), using the same conditions and column described above. The column was related to the ion source of the mass spectrometer. Mass units were monitored from 10 to 900 at 70 eV. The identification of compounds was based on a comparison of their retention times with those of authentic samples and/or by comparison of their mass spectra with those of published data [22,49,50].

### 4.5. Methanolic Extracts 

The vegetable biomass deriving from the various accessions was cold extracted using methanol acidified with 0.1% HCl (37%). The fresh aerial plant parts (100 g) were treated with a grinder and then subjected to maceration three times with 300 mL of acidified CH_3_OH at room temperature in a flask with a magnetic stir bar at 400 rpm (30 h). At the end of this period, the resulting solution was dried by evaporating the solvent under vacuum, taking care that the temperature of the water bath did not exceed 50 °C. The residue was dissolved with 250 mL of distilled water and extracted three times with portions of 100 mL of CHCl_3_. The solvent was evaporated under vacuum at room temperature, and the residue was used for subsequent analyzes. The yields of extracts varied between 1.5% and 2.4% (Table 5).

### 4.6. GC/MS Analyses

MS analyses were carried out on the extracts (Section 4.5) according to Peroutka et al. [44] with some modification. Briefly, we used an Agilent Technologies model 7820A connected with an MS detector 5977E MSD (Agilent Technologies Italia S.p.A, Milano, Italy), along with a 60 m × 0.25 mm, thickness 0.25 μm ZB-5 ms fused silica capillary column (Phenomenex). The injection port and detector temperatures were 280 °C. The samples (0.1 μL each) were injected using a split/splitless automatic injector HP 7673 with helium (1.0 mL/min) as a carrier gas. Temperature program conditions were as follows: the initial temperature was set at 70 °C, ramped up to 230 °C at 20 °C/min, and then ramped up to 250 °C at 5 °C/min. The column was related to the ion source of the mass spectrometer. Mass units were monitored from 10 to 900 at 70 eV. The quantification of furocoumarins was conducted using the addition method [51] with the necessary variations, in almost all areas of chemical analyses. To avoid excessive errors, the added analyte concentrations were of the same magnitude as those already present in the samples. To not substantially change the solution, we started from a very concentrated standard solution to add minimum volumes. The study of the corresponding variation of the signal obtained allowed us to determine the concentration in the sample, expressed as mg/100 g of fresh plant (Figure 1).

### 4.7. NMR Analysis 

Psoralens were isolated as described in the literature [7] from the extract of *B. b. var bituminosa* (accession Monte Rosello, SS) and from the extract of *B. morisiana* (accession Monte Gonareddu). 

One gram of the crude extract of *B. b.* var. *bituminosa* derived from the Monte Rosello accession was suspended in *n*-hexane and placed at the top of a silica gel column (20 mm × 2.5 mm, Kieselgel 60, 0.015–0.040 mm), before eluting with *n*-hexane/Et_2_O at different ratios with increasing Et_2_O concentration. All collected fractions were analyzed by TLC, by elution with *n*-hexane/Et_2_O (1:1 *v*/*v*). Pure fractions containing angelicin (40 mg) and psoralen (5 mg) were obtained. 

The same procedure was applied to 1 g of crude extract of *B. morisiana* derived from the Monte Gonareddu accession; in this case, we obtained 8 mg of angelicin and 19 mg of psoralen.

The identity of these compounds was assessed by ^1^H and ^13^C nuclear magnetic resonance (NMR), using a VARIAN (Mercury plus) spectrometer operating at 399.93 MHz for ^1^H and 100.57 MHz for ^13^C, with the sample dissolved in CDCl_3_, using tetramethylsilane (TMS) as an internal reference.

Angelicin: ^1^H-NMR (400 MHz, CHCl_3_), *δ* ppm: 7.83 (1H, *d,* J = 9.6 Hz); 7.71 (1H, d, J = 2.4 Hz); 7.45 (1H, *d,* J = 8.8 Hz); 7.40 (1H, *d,* J = 8.8 Hz); 7.15 (1H, *d,* J = 2.4 Hz); 6.41 (H, *d,* J = 9.6 Hz). ^13^C-NMR (100 MHz, CHCl_3_), *δ* ppm: 160.66 (C-2), 157.33 (C-7), 148.93 (C-9), 145.90 (C-12), 144.59 (C-4), 141.25 (C-3), 123.82 (C-5), 119.82 (C-5), 117.04 (C-8), 114.10 (C-10), 108.84 (C-6), 104.11 (C-11).

Psolaren: ^1^H-NMR (400 MHz, CHCl_3_), *δ* ppm: 7.82 (1H, *d,* J = 10 Hz); 7.71 (1H, *d,* J = 2.4 Hz); 7.70 (1H, s); 6.84 (1H, *d,* J = 2.4 Hz); 6.39 (H, *d,* J = 10 Hz). ^13^C-NMR (100 MHz, CHCl_3_), *δ* ppm: 161.10 (C-2), 156.37 (C-7), 151.96 (C-9), 146.91 (C-12), 144.12 (C-4), 124.86 (C-6), 119.82 (C-5), 115.37 (C-10), 114.60 (C-3), 106.35 (C-11), 99.87 (C-8).

### 4.8. Statistical Analysis

Data analyses of three replicates were performed for every sample. ANOVA was applied with a factorial design (MSTAT-C, software developed by the Crop and Soil Sciences Department of Michigan State University of the United States). Mean separation was tested by application of Tukey’s test. To investigate chemical variation in the 15 different accessions of *Bituminaria* based on GC and GC/MS, we submitted the data to multivariate statistical evaluation (PCA). PCA is an unsupervised pattern recognition technique that converts data consisting of many interrelated variables to a new coordinate system, thereby reducing dimensionality while maintaining the variance [52]. PCA reveals trends in a dataset such as groupings and clusters based on chemical similarities or differences, while outliers within the dataset are also identified. The results of PCA were observed in a score scatter plot, displaying the spatial distribution of observations. Prior to chemometric analysis, the total integral areas were set to 100 to normalize the data, and the generated ASCII file was imported into Microsoft Excel for labeling. The matrix was then imported into SIMCA-P software version 12.0 (Umetrics AB, Umea, Sweden) for statistical analysis.

## 5. Conclusions

The aim of the present research was to discriminate *Bituminaria* populations with low furocoumarin content, useful for fodder production in Mediterranean environments, or *Bituminaria* populations with high furocoumarin content, useful for pharmaceutical purposes. For this reason, we characterized the volatile compounds of 15 different accessions of native Sardinian populations of *B. morisiana* and *B. bituminosa* and Spanish native accessions of *B. bituminosa* and its varieties. We demonstrated that it is not easy recognize the various plants belonging to the genus *Bituminaria* using secondary metabolites, because most represented constituents are often the same (although in different concentrations); however, considering all characterized volatile compounds in our work and carrying out a principal component analysis, it was possible to clearly distinguish the species *B. morisiana* and *B. bituminosa*. Moreover, using only components of the essential oils, we also evidenced the differences between *B. b.* var. *crassiuscula* and the other *B. bituminosa* varieties, as well as *B. morisiana*, as reported in the score plot of PCA analysis performed using the essential oil components, where a clear differentiation of the varieties *B. b.* var. *crassiuscula* and *B. morisiana* from *B. b.* var. *bituminosa* and *B. b.* var. *Albomarginata* is shown.

## Figures and Tables

**Table 1:** Chemical composition of *Bituminaria bituminosa* (L.) C.H. Stirt. essential oils from Sardinia and Spain genotypes grown in a Sardinian experimental field.

			*Bituminaria bituminosa* var. *bituminosa* Sardinia			*Bituminaria bituminosa* var. *bituminosa* Spain				
RI Lett Apolar	RI Exp Apolar	Compounds	*B. b.* Siniscola	*B. b.* Loculi	*B. b*. SS	*B. b.* Llano del Beal	*B. b.* Calnegre	*B. b.* San Cristòbal de la Laguna	ID *	References
921	927	tricyclene							Std	
939	937	α-pinene							Std	
955	954	camphene							Std	
992	991	β-myrcene							Std	
1031	1029	limonene							Std	
1031	1030	β-fellandrene							Std	
1052	1050	*trans*-β-ocimene		0.41 ± 0.05		1.04 ± 0.09			Std	
1282	1275	citronellyl formate		0.28 ± 0.02	0.21 ± 0.01	1.07 ± 0.10			MS-RI	[29]
1320	1317	2,4-dodecadienal							MS-RI	
1323	1326	methyl geraniate							MS-RI	
1345	1351	α-cubebene	0.26 ± 0.01	0.26 ± 0.02	0.21 ± 0.01	0.58 ± 0.04			Std	
1373	1375	α-ylangene							Std	
1374	1377	α-copaene	0.97 ± 0.02	0.64 ± 0.02	0.56 ± 0.03		0.55 ± 0.04	3.15 ± 0.22	Std	
1385	1385	*trans*-β-damascenone	0.23 ± 0.01	0.43 ± 0.02	0.54 ± 0.02	0.32 ± 0.02	0.22 ± 0.01	0.63 ± 0.4	MS-RI	[30]
1387	1387	β-bourbonene	1.68 ± 0.09	1.64 ± 0.07	1.34 ± 0.08	0.51 ± 0.02	0.70 ± 0.02	1.94 ± 0.22	Std	
1388	1388	farnesene isomer							MS	
1387	1390	β-cubebene	0.38 ± 0.02						Std	
1389	1393	β-elemene	0.42 ± 0.02			0.34 ± 0.01	0.43 ± 0.02	0.74 ± 0.07	Std	
1396	1396	1,5,8-trimethyl-1,2-dihydronaphthalene		0.60 ± 0.04	0.31 ± 0.04				MS	
1411	1409	isocaryophyllene							MS-RI	[31]
1412	1412	1,2-dihydro-1,4,6-trimethylnaphthalene		0.44 ± 0.03	0.63 ± 0.04				MS	
1418	1418	dihydrodehydro-β-ionone		0.72 ± 0.06			0.40 ± 0.02		MS	
1419	1419	β-caryophyllene	15.8 ± 0.32	18.96 ± 0.37	17.31 ± 0.33	34.47 ± 0.62	14.23 ± 0.35	8.18 ± 0.34	Std	
1419	1419	β-cedrene	1.55 ± 0.07	1.49 ± 0.09		0.64 ± 0.05			Std	
1430	1433	*epi*-bicyclosesquiphellandrene			1.94 ± 0.011		0.95 ± 0.12	1.50 ± 0.05	MS-RI	[32]
1431	1433	β-gurjunene	0.33 ± 0.01	0.39 ± 0.03	0.27 ± 0.01	0.15 ± 0.01		0.31 ± 0.04	Std	
1439	1441	aromadendrene							Std	
1434	1437	γ-elemene							Std	
1440	1443	*cis*-β-farnesene	3.07 ± 0.10	1.49 ± 0.08	5.74 ± 0.07	8.19 ± 0.31	9.43 ± 0.29	3.15 ± 0.11	Std	
1452	1451	α-humulene	3.36 ± 0.09	3.83 ± 0.11	4.11 ± 0.14	7.75 ± 0.13	3.83 ± 0.18	2.75 ± 0.09	Std	
1452	1452	α-himachalene							MS-RI	
1458	1460	alloaromadendrene	1.47 ± 0.04	0.90 ± 0.05	1.04 ± 0.09	0.58 ± 0.04	0.80 ± 0.05	2.66 ± 0.14	MS-RI	
1473	1471	lauryl alcohol			0.25 ± 0.01				MS-RI	[33]
1478	1480	γ-muurolene	2.16 ± 0.09	1.87 ± 0.21	1.69 ± 0.19	0.69 ± 0.04	1.51 ± 0.04	2.37 ± 0.08	Std	
1484	1485	germacrene D	10.46 ± 0.28	5.38 ± 0.35	8.62 ± 0.27	2.2 ± 0.08	4.56 ± 0.12	5.48 ± 0.31	Std	
1493	1493	*epi*-cubebol	1.2 ± 0.05	0.93 ± 0.12	0.93 ± 0.09		0.68 ± 0.07	1.14 ± 0.07	MS-RI	
1493	1494	α-zingiberene							MS-RI	
1500	1500	α-muurolene	1.30 ± 0.04	0.98 ± 0.09	1.07 ± 0.12		0.52 ± 0.04	1.36 ± 0.07	Std	
1505	1509	β-bisabolene			0.10 ± 0.01		0.11 ± 0.02		Std	
1508	1510	α-farnesene	0.51 ± 0.03	0.31 ± 0.02					Std	
1513	1514	γ-cadinene	0.70 ± 0.04	0.78 ± 0.03	0.65 ± 0.02		0.31 ± 0.02	0.55 ± 0.04	Std	
1514	1519	cubebol	0.69 ± 0.07	0.45 ± 0.02	0.64 ± 0.02			1.26 ± 0.11	MS-RI	
1520	1522	7-epi-α-selinene							MS-RI	
1521	1523	β-sesquiphellandrene							MS-RI	
1522	1523	δ-cadinene	2.10 ± 0.11	1.82 ± 0.14	1.76 ± 0.08	0.79 ± 0.02	1.08 ± 0.08	3.42 ± 0.31	Std	
1539	1539	α-copaen-11-ol							MS-RI	
1548	1550	elemol							Std	
1559	1561	germacrene B							MS-RI	
1565	1564	*cis*-nerolidol	3.74 ± 0.15	0.33 ± 0.04	0.41 ± 0.02	0.44 ± 0.01	0.84 ± 0.05	0.32 ± 0.02	Std	
1569	1567	*cis*-3-hexenyl benzoate	0.48 ± 0.04	0.24 ± 0.02	0.25 ± 0.01	0.53 ± 0.04			MS-RI	[34]
1574	1575	germacrene-4-ol	0.27 ± 0.02	0.39 ± 0.02	0.24 ± 0.01		0.23 ± 0.01	0.80 ± 0.04	MS-RI	
1577	1578	spathulenol	0.62 ± 0.04	0.51 ± 0.03	0.62 ± 0.04		0.17 ± 0.01	0.51 ± 0.02	MS-RI	
1582	1583	caryophyllene oxide	13.98 ± 0.25	19.56 ± 0.44	14.24 ± 0.37	18.5 ± 0.28	7.63 ± 0.24	5.58 ± 0.22	Std	
1589	1586	isocaryophyllene	0.97 ± 0.08	0.91 ± 0.06	1.11 ± 0.14	0.74 ± 0.04	0.21 ± 0.02	0.77 ± 0.09	MS-RI	
1590	1587	globulol	0.39 ± 0.02	0.38 ± 0.01	0.29 ± 0.01				Std	
1592	1593	cedrol			0.58 ± 0.04				MS-RI	
1592	1594	viridiflorene	0.64 ± 0.03				0.65 ± 0.04		MS-RI	
1608	1601	α-humulene-epoxide II	1.84 ± 0.06	2.63 ± 0.09	2.11 ± 0.13	2.77 ± 0.17	1.42 ± 0.09	1.29 ± 0.11	MS-RI	
1618	1614	1,10-di-epi-cubebol	0.27 ± 0.01		0.33 ± 0.01		0.22 ± 0.01		MS-RI	
1618	1619	*cis*-bisabol-11-ol	0.51 ± 0.01			0.56 ± 0.04			MS-RI	
1620	1622	muurola-4,10(14)-dien-1β-ol	0.29 ± 0.02	0.51 ± 0.07		0.36 ± 0.04			MS-RI	
1629	1627	1-epi-cubenol	0.71 ± 0.04		0.69 ± 0.03		0.44 ± 0.04	1.34 ± 0.09	MS-RI	
1630	1630	longifolene aldehyde	0.44 ± 0.03						MS	
1630	1632	γ-eudesmol		0.36 ± 0.02	0.41 ± 0.02			2.52 ± 0.22	Std	
1631	1641	caryophylla-4(12),8(13)-dien-5α-ol		0.52 ± 0.03	0.41 ± 0.02	0.57 ± 0.04	0.38 ± 0.04		MS-RI	[35]
1637	1641	caryophylla-4(14),8(15)-dien-5β-ol	2.26 ± 0.13	2.30 ± 0.11	1.44 ± 0.09	1.70 ± 0.14	0.29 ± 0.02		MS-RI	[36]
1644	1646	τ-muurolol					1.61 ± 0.33		MS-RI	
1645	1647	cubenol	0.63 ± 0.04	0.63 ± 0.04	0.82 ± 0.08		0.52 ± 0.04	0.81 ± 0.05	Std	
1652	1653	α-cadinol	2.71 ± 0.09	2.53 ± 0.10	3.34 ± 0.28	0.58 ± 0.04	2.23 ± 0.09	3.31 ± 0.35	Std	
1674	1672	β-bisabolol							Std	
1675	1677	*n*-tetradecanol	0.61 ± 0.04	0.75 ± 0.04	0.36 ± 0.04	3.37 ± 0.29	0.31 ± 0.02	2.57 ± 0.22	MS-RI	
1678	1678	aromadendrene-oxide 2	1.44 ± 0.07	1.97 ± 0.05	1.25 ± 0.06	1.48 ± 0.11	0.72 ± 0.04	0.49 ± 0.04	MS-RI	[37]
1682	1682	ledene oxide II	2.12 ± 0.05	1.37 ± 0.07	2.31 ± 0.07		1.02 ± 0.08	1.70 ± 0.15	MS-RI	[38]
1704	1704	bisabolene oxide							MS-RI	
1718	1721	*cis*,*cis*-2,6-farnesol			0.47 ± 0.04		0.94 ± 0.05		MS-RI	[39]
1756	1757	myristic acid							MS-RI	
1760	1762	benzyl benzoate	0.19 ± 0.01	0.29 ± 0.01	0.29 ± 0.01		0.34 ± 0.02	0.45 ± 0.02	MS-RI	
1774	1781	1-pentadecanol							MS-RI	
1792	1792	1,2-15,16-diepoxy-hexadecane	0.33 ± 0.02	0.25 ± 0.01			0.98 ± 0.04	0.77 ± 0.04	MS	
1810	1816	*cis*-11-hexadecenal	0.55 ± 0.01				0.90 ± 0.04	0.48 ± 0.02	MS-RI	
1827	1827	benzyl salicylate							MS-RI	[40]
1863	1864	1-hexadecanol					1.44 ± 0.09		MS	
1863	1866	*cis*-9-hexadecen-1-ol					0.25 ± 0.02		MS-RI	
1890	1890	2-methylhexadecan-1-ol	0.68 ± 0.04	0.99 ± 0.02					MS	
1946	1944	palmitic acid	1.29 ± 0.05	1.11 ± 0.09	0.93 ± 0.06	0.59 ± 0.04	1.15 ± 0.17	2.65 ± 0.27	MS-RI	
2046	2046	geranyl linalool				0.45 ± 0.04			MS	
2052	2052	*cis*-*cis*-9,12-octadecadien-1-ol	1.85 ± 0.14	3.87 ± 0.42	5.28 ± 0.41	0.69 ± 0.06	15.76 ± 0.29	11.06 ± 0.17	MS-RI	[22]
2058	2058	*cis*-*cis*-*cis*-9,12,15-octadecatrien-1-ol	5.2 ± 0.31	4.97 ± 0.044	4.06 ± 0.27	0.34 ± 0.02	6.92 ± 0.17	6.58 ± 0.20	MS-RI	[41]
2060	2060	*cis*-9-octadecen-1-ol							MS-RI	
2074	2074	*n*-octadecyl alcohol							MS-RI	
2104	2104	12-methyl-*E*,*E*-2,13-octadecadien-1-ol	0.19 ± 0.01					0.47 ± 0.04	MS-RI	
2114	2114	phytol	4.25 ± 0.25	3.02 ± 0.33	1.12 ± 0.9	1.66 ± 0.13	4.88 ± 0.06	3.95 ± 0.25	MS-RI	
2178	2178	linolenic acid		0.45 ± 0.04	0.23 ± 0.01	0.65 ± 0.04		0.11 ± 0.01	MS-RI	
2279	2279	methyl 11,14,17-icosatrienoate		0.81 ± 0.04	0.52 ± 0.04	0.38 ± 0.02		2.33 ± 0.13	MS-RI	
2241	2241	*trans*-*trans*-*cis*-1,3,12-nonadecatriene-5,14-diol							MS-RI	
		No. of identified constituents	49	48	49	34	44	40		
			98.09	96.65	94.03	95.68	92.76	91.45		

* ID = identification methods: MS by comparison of the mass spectrum with those of the computer mass libraries Adams and Nist 11, and by interpretation of the mass spectral fragmentations; RI by comparison of retention index with those reported in the literature; Std by comparison of the retention time and mass spectrum of available authentic standards; MS by identification of the mass spectrum. Nonpolar column ZB-5. Data are the means of three replicates.

**Table 2:** Chemical composition of *B. bituminosa* var. *albomarginata* and *B. bituminosa* var. *crassiuscula* essential oils from Spain genotypes growing up in a Sardinian experimental field.

			*Bituminaria bituminosa* var. *albomarginata*		*Bituminaria bituminosa* var. *crassiuscula*			
RI Lett Apolar	RI Exp Apolar	Compounds	*B. b.* var. *albomarginata* Caleta de Famara	*B. b.* var. *albomarginata* Arecife	*B. b.* var. *crassiuscula* San Cristòbal de la Laguna	*B. b.* var. *crassiuscula* Vilaflor	ID *	References
921	927	tricyclene					Std	
939	937	α-pinene					Std	
955	954	camphene					Std	
992	991	β-myrcene			0.37 ± 0.04		Std	
1031	1029	limonene					Std	
1031	1030	β-fellandrene					Std	
1052	1050	*trans*-β-ocimene		0.13 ± 0.01			Std	
1282	1275	citronellyl formate		0.16 ± 0.01			MS-RI	[29]
1320	1317	2,4-dodecadienal				0.78 ± 0.09	MS-RI	
1323	1326	methyl geraniate			0.50 ± 0.08		MS-RI	
1345	1351	α-cubebene		0.28 ± 0.01			Std	
1373	1375	α-ylangene					Std	
1374	1377	α-copaene	1.47 ± 0.12	3.19 ± 0.15	1.50 ± 0.11	0.89 ± 0.08	Std	
1385	1385	*trans*-β-damascenone	0.25 ± 0.01			0.35 ± 0.04	MS-RI	[30]
1387	1387	β-bourbonene	1.43 ± 0.05	0.82 ± 0.11	0.46 ± 0.05		Std	
1388	1388	farnesene isomer					MS	
1387	1390	β-cubebene					Std	
1389	1393	β-elemene	0.39 ± 0.02	0.96 ± 0.11	0.74 ± 0.04	0.44 ± 0.04	Std	
1396	1396	1,5,8-trimethyl-1,2-dihydronaphthalene					MS	
1411	1409	isocaryophyllene				0.42 ± 0.04	MS-RI	[31]
1412	1412	1,2-dihydro-1,4,6-trimethylnaphthalene					MS	
1418	1418	dihydrodehydro-β-ionone					MS	
1419	1419	β-caryophyllene	7.62 ± 0.22	4.32 ± 0.24	21.98 ± 0.48	24.89 ± 0.51	Std	
1419	1419	β-cedrene	0.91 ± 0.05	1.10 ± 0.08	0.44 ± 0.04	0.53 ± 0.04	Std	
1430	1433	*epi*-bicyclosesquiphellandrene					MS-RI	[32]
1431	1433	β-gurjunene	0.40 ± 0.02				Std	
1439	1441	aromadendrene					Std	
1434	1437	γ-elemene			0.51 ± 0.04		Std	
1440	1443	*cis*-β-farnesene	0.61 ± 0.04	2.97 ± 0.33		0.53 ± 0.04	Std	
1452	1451	α-humulene	2.97 ± 0.17	1.56 ± 0.18	7.53 ± 0.24	4.92 ± 0.41	Std	
1452	1452	α-himachalene			0.57 ± 0.04		MS-RI	
1458	1460	alloaromadendrene	2.16 ± 0.12		0.99 ± 0.06	0.78 ± 0.05	MS-RI	
1473	1471	lauryl alcohol		13.83 ± 0.45			MS-RI	[33]
1478	1480	γ-muurolene	2.07 ± 0.09	3.14 ± 0.22	1.52 ± 0.33	0.46 ± 0.04	Std	
1479	1483	α-curcumene			0.83 ± 0.12	0.21 ± 0.02	MS-RI	
1484	1485	germacrene D	8.45 ± 0.32	12.40 ± 0.20	2.97 ± 0.32	1.72 ± 0.33	Std	
1493	1493	*epi*-cubebol	1.18 ± 0.08	0.66 ± 0.12	0.81 ± 0.08	0.45 ± 0.02	MS-RI	
1493	1494	α-zingiberene					MS-RI	
1500	1500	α-muurolene	1.17 ± 0.10	2.08 ± 0.09	0.88 ± 0.04	0.23 ± 0.01	Std	
1505	1509	β-bisabolene			0.38 ± 0.02		Std	
1508	1510	α-farnesene					Std	
1513	1514	γ-cadinene		0.39 ± 0.04	1.49 ± 0.22	0.21 ± 0.01	Std	
1514	1519	cubebol	1.91 ± 0.22		0.95 ± 0.06	0.86 ± 0.05	MS-RI	
1520	1522	7-*epi*-α-selinene		0.76 ± 0.04			MS-RI	
1521	1523	β-sesquiphellandrene		2.05 ± 0.15			MS-RI	
1522	1523	δ-cadinene	2.96 ± 0.33	2.71 ± 0.31	0.59 ± 0.04	1.22 ± 0.21	Std	
1539	1539	α-copaen-11-ol		0.55 ± 0.05		0.24 ± 0.01	MS-RI	
1548	1550	elemol			0.59 ± 0.04		Std	
1559	1561	germacrene B	0.42 ± 0.04	0.35 ± 0.02			MS-RI	
1565	1564	*cis*-nerolidol		0.22 ± 0.02			Std	
1569	1567	*cis*-3-hexenyl benzoate			1.12 ± 0.11		MS-RI	[34]
1574	1575	germacrene-4-ol		0.63 ± 0.04			MS-RI	
1577	1578	spathulenol	0.43 ± 0.04	0.44 ± 0.04			MS-RI	
1582	1583	caryophyllene oxide	3.50 ± 0.25	1.44 ± 0.11	7.43 ± 0.23	11.02 ± 0.24	Std	
1589	1586	isocaryophyllene					MS-RI	
1590	1587	globulol			0.98 ± 0.13	0.75 ± 0.07	Std	
1592	1593	cedrol					MS-RI	
1592	1594	viridiflorene	2.37 ± 0.19	0.81 ± 0.04			MS-RI	
1608	1601	α-humulene-epoxide II	1.04 ± 0.12	0.56 ± 0.03	1.91 ± 0.14	1.35 ± 0.33	MS-RI	
1618	1614	1,10-di-*epi*-cubebol	0.95 ± 0.08	1.21 ± 0.11	0.68 ± 0.08	0.42 ± 0.04	MS-RI	
1618	1619	*cis*-bisabol-11-ol					MS-RI	
1620	1622	muurola-4,10(14)-dien-1β-ol					MS-RI	
1629	1627	1-*epi*-cubenol					MS-RI	
1630	1630	longifolene aldehyde					MS	
1630	1632	γ-eudesmol					Std	
1631	1641	caryophylla-4(12),8(13)-dien-5α-ol			0.69 ± 0.06	0.74 ± 0.08	MS-RI	[35]
1637	1641	caryophylla-4(14),8(15)-dien-5β-ol			0.95 ± 0.05	2.20 ± 0.17	MS-RI	[36]
1644	1646	τ-muurolol					MS-RI	
1645	1647	cubenol	0.96 ± 0.07				Std	
1652	1653	α-cadinol	3.95 ± 0.37	3.14 ± 0.13	1.63 ± 0.11	0.94 ± 0.09	Std	
1674	1672	β-bisabolol			0.32 ± 0.02		Std	
1675	1677	*n*-tetradecanol	0.78 ± 0.08	10.93 ± 0.51			MS-RI	
1678	1678	aromadendrene oxide 2			0.39 ± 0.02	1.01 ± 0.08	MS-RI	[37]
1682	1682	ledene oxide II	2.02 ± 0.13	1.48 ± 0.18	0.68 ± 0.04	0.43 ± 0.04	MS-RI	[38]
1704	1704	bisabolene oxide					MS-RI	
1718	1721	*cis*,*cis*-2,6-farnesol					MS-RI	[39]
1756	1757	myristic acid			0.31 ± 0.02	0.48 ± 0.04	MS-RI	
1760	1762	benzyl benzoate	0.82 ± 0.08		3.05 ± 0.22	2.38 ± 0.34	MS-RI	
1774	1781	1-pentadecanol					MS-RI	
1792	1792	1,2-15,16-diepoxy-hexadecane	0.73 ± 0.04	0.67 ± 0.04	0.84 ± 0.10	0.86 ± 0.13	MS	
1810	1816	*cis*-11-hexadecenal	1.19 ± 0.06	0.73 ± 0.05	0.99 ± 0.11	1.01 ± 0.07	MS-RI	
1827	1827	benzyl salicylate			3.45 ± 0.31	3.51 ± 0.34	MS-RI	[40]
1863	1864	1-hexadecanol					MS	
1863	1866	*cis*-9-hexadecen-1-ol					MS-RI	
1890	1890	2-methylhexadecan-1-ol	1.54 ± 0.13	0.87 ± 0.04			MS	
1946	1944	palmitic acid	4.23 ± 0.22	1.12 ± 0.11	2.37 ± 0.26	1.61 ± 0.22	MS-RI	
2046	2046	geranyl linalool			0.58 ± 0.04		MS	
2052	2052	*cis-cis*-9,12-octadecadien-1-ol	14.96 ± 0.40	5.64 ± 0.24	11.54 ± 0.38	12.03 ± 0.41	MS-RI	[22]
2058	2058	*cis-cis-cis*-9,12,15-octadecatrien-1-ol	9.86 ± 0.21	4.83 ± 0.31	6.71 ± 0.24	7.33 ± 0.31	MS-RI	[41]
2060	2060	*cis*-9-octadecen-1-ol					MS-RI	
2074	2074	*n*-octadecyl alcohol					MS-RI	
2104	2104	12-methyl-*E*,*E*-2,13-octadecadien-1-ol	0.51 ± 0.04				MS-RI	
2114	2114	phytol	4.89 ± 0.19	3.18 ± 0.18	2.76 ± 0.18	5.13 ± 0.27	MS-RI	
2178	2178	linolenic acid	2.72 ± 0.08		1.19 ± 0.14	1.14 ± 0.12	MS-RI	
2279	2279	methyl 11,14,17-icosatrienoate					MS-RI	
2241	2241	*trans-trans-cis*-1,3,12-nonadecatriene-5,14-diol		0.40 ± 0.11			MS-RI	
		No. of identified constituents	36	39	43	38		
			93.82	92.71	97.17	94.47		

* ID = identification methods: MS by comparison of the mass spectrum with those of the computer mass libraries Adams and Nist 11, and by interpretation of the mass spectral fragmentations; RI by comparison of the retention index with those reported in the literature; Std by comparison of the retention time and mass spectrum of available authentic standards; MS identification of the mass spectrum. Nonpolar column ZB-5. Data are the mean of three replicates.

**Table 3:** Chemical composition of Sardinian endemic *Bituminaria morisiana* (Pignatti & Metlesics) Greuter essential oils growing up in a Sardinian experimental field.

			*Bituminaria Morisiana*						
RI Lett Apolar	RI Exp Apolar	Compounds	Punta Giglio	Burcei	Bitti	Siliqua	Monte Gonareddu	ID *	References
921	927	tricyclene				0.17 ± 0.01		Std	
939	937	α-pinene				0.67 ± 0.02		Std	
955	954	camphene				0.39 ± 0.01		Std	
992	991	β-myrcene				0.72 ± 0.04		Std	
1031	1029	limonene				0.35 ± 0.02		Std	
1031	1030	β-fellandrene				0.26 ± 0.01		Std	
1052	1050	*trans*-β-ocimene						Std	
1282	1275	citronellyl formate						MS-RI	[29]
1320	1317	2,4-dodecadienal						MS-RI	
1323	1326	methyl geraniate						MS-RI	
1345	1351	α-cubebene		0.39 ± 0.01	0.31 ± 0.01	0.43 ± 0.02		Std	
1373	1375	α-ylangene		0.20 ± 0.01	0.18 ± 0.01			Std	
1374	1377	α-copaene	0.43 ± 0.02	0.20 ± 0.01	0.55 ± 0.02	0.35 ± 0.02	0.65 ± 0.02	Std	
1385	1385	*trans*-β-damascenone						MS-RI	[30]
1387	1387	β-bourbonene	0.42 ± 0.04	0.84 ± 0.04	0.74 ± 0.03	0.35 ± 0.02	0.92 ± 0.05	Std	
1388	1388	farnesene isomer	0.21 ± 0.01	0.30 ± 0.02				MS	
1387	1390	β-cubebene	0.40 ± 0.02		0.08 ± 0.01			Std	
1389	1393	β-elemene						Std	
1396	1396	1,5,8-trimethyl-1,2-dihydronaphthalene						MS	
1411	1409	isocaryophyllene						MS-RI	[31]
1412	1412	1,2-dihydro-1,4,6-trimethylnaphthalene						MS	
1418	1418	dihydrodehydro-β-ionone						MS	
1419	1419	β-caryophyllene	0.62 ± 0.04	2.91 ± 0.11	9.92 ± 0.29	9.06 ± 0.26	4.54 ± 0.19	Std	
1419	1419	β-cedrene	1.41 ± 0.09	0.76 ± 0.05		0.56 ± 0.04		Std	
1430	1433	*epi*-bicyclosesquiphellandrene						MS-RI	[32]
1431	1433	β-gurjunene	0.61 ± 0.04		0.80 ± 0.04			Std	
1439	1441	aromadendrene		0.18 ± 0.01	0.20 ± 0.01			Std	
1434	1437	γ-elemene						Std	
1440	1443	*cis*-β-farnesene	41.77 ± 0.42	34.36 ± 0.37	26.91 ± 0.28	35.11 ± 0.32	37.01 ± 0.28	Std	
1452	1451	α-humulene						Std	
1452	1452	α-himachalene						MS-RI	
1458	1460	alloaromadendrene	0.68 ± 0.04		1.40 ± 0.11	0.84 ± 0.06	0.76 ± 0.04	MS-RI	
1473	1471	lauryl alcohol						MS-RI	[33]
1478	1480	γ-muurolene	1.20 ± 0.13	2.13 ± 0.24	2.00 ± 0.19	1.35 ± 0.07	1.66 ± 0.33	Std	
1479	1483	α-curcumene						MS-RI	
1484	1485	germacrene D	2.84 ± 0.36	3.41 ± 0.38	3.26 ± 0.16	3.81 ± 0.37	4.95 ± 0.42	Std	
1493	1493	*epi*-cubebol	0.94 ± 0.11	1.54 ± 0.21	1.24 ± 0.08	1.11 ± 0.10	1.38 ± 0.14	MS-RI	
1493	1494	α-zingiberene	2.08 ± 0.24	1.73 ± 0.19	1.50 ± 0.06	1.74 ± 0.22	1.25 ± 0.11	MS-RI	
1500	1500	α-muurolene	0.88 ± 0.09	1.22 ± 0.09	0.83 ± 0.04	1.29 ± 0.16	0.90 ± 0.08	Std	
1505	1509	β-bisabolene						Std	
1508	1510	α-farnesene	0.42 ± 0.04	0.41 ± 0.04	0.25 ± 0.02	0.32 ± 0.04		Std	
1513	1514	γ-cadinene	0.65 ± 0.04	0.95 ± 0.08	0.81 ± 0.04	0.63 ± 0.04	0.90 ± 0.08	Std	
1514	1519	cubebol	0.57 ± 0.04	0.75 ± 0.04	0.65 ± 0.04	0.76 ± 0.05	0.76 ± 0.04	MS-RI	
1520	1522	7-epi-α-selinene						MS-RI	
1521	1523	β-sesquiphellandrene						MS-RI	
1522	1523	δ-cadinene	1.72 ± 0.19	2.85 ± 0.38	2.22 ± 0.31	2.27 ± 0.25	2.59 ± 0.33	Std	
1539	1539	α-copaen-11-ol		0.46 ± 0.04		0.33 ± 0.05		MS-RI	
1548	1550	elemol			0.21 ± 0.01			Std	
1559	1561	germacrene B			0.21 ± 0.01			MS-RI	
1565	1564	*cis*-nerolidol	1.94 ± 0.22	1.90 ± 0.12	1.14 ± 0.12	1.66 ± 0.10	1.32 ± 0.11	Std	
1569	1567	*cis*-3-hexenyl benzoate						MS-RI	[34]
1574	1575	germacrene-4-ol	0.56 ± 0.05	1.23 ± 0.11	0.43 ± 0.02	1.09 ± 0.10		MS-RI	
1577	1578	spathulenol	0.66 ± 0.04	1.10 ± 0.6	0.60 ± 0.02	0.71 ± 0.06	0.59 ± 0.09	MS-RI	
1582	1583	caryophyllene oxide		1.44 ± 0.16	2.81 ± 0.40	2.34 ± 0.22	1.80 ± 0.14	Std	
1589	1586	isocaryophyllene						MS-RI	
1590	1587	globulol	0.63 ± 0.04					Std	
1592	1593	cedrol						MS-RI	
1592	1594	viridiflorene	0.70 ± 0.13	0.70 ± 0.05	0.55 ± 0.02	0.38 ± 0.02		MS-RI	
1608	1601	α-humulene-epoxide II		0.47 ± 0.04	0.61 ± 0.02	0.34 ± 0.02		MS-RI	
1618	1614	1,10-di-epi-cubebol	0.41 ± 0.05	0.78 ± 0.04	0.70 ± 0.04	0.43 ± 0.02	0.82 ± 0.04	MS-RI	
1618	1619	*cis*-bisabol-11-ol	1.13 ± 0.14	1.13 ± 0.12	1.04 ± 0.12	0.82 ± 0.03		MS-RI	
1620	1622	muurola-4,10(14)-dien-1β-ol						MS-RI	
1629	1627	1-*epi*-cubenol		0.59 ± 0.04			0.68 ± 0.04	MS-RI	
1630	1630	longifolene aldehyde						MS	
1630	1632	γ-eudesmol						Std	
1631	1641	caryophylla-4(12),8(13)-dien-5α-ol						MS-RI	[35]
1637	1641	caryophylla-4(14),8(15)-dien-5β-ol						MS-RI	[36]
1644	1646	τ-muurolol	1.61 ± 0.16	2.13 ± 0.31	1.77 ± 0.35	2.89 ± 0.15	2.46 ± 0.13	MS-RI	
1645	1647	cubenol	0.41 ± 0.04	0.47 ± 0.04	0.64 ± 0.07	0.65 ± 0.09		Std	
1652	1653	α-cadinol	1.87 ± 0.31	2.56 ± 0.14	2.77 ± 0.10	2.14 ± 0.22	3.24 ± 0.16	Std	
1674	1672	β-bisabolol						Std	
1675	1677	*n*-tetradecanol						MS-RI	
1678	1678	aromadendrene-oxide 2				0.62 ± 0.04		MS-RI	[37]
1682	1682	ledene oxide II	0.30 ± 0.02	0.83 ± 0.04	0.87 ± 0.04		1.10 ± 0.10	MS-RI	[38]
1704	1704	bisabolene oxide	0.50 ± 0.04	0.71 ± 0.04	0.20 ± 0.02			MS-RI	
1718	1721	*cis*,*cis*-2,6-farnesol	1.76 ± 0.21	1.26 ± 0.09	0.87 ± 0.04	1.04 ± 0.10	1.22 ± 0.11	MS-RI	[39]
1756	1757	myristic acid						MS-RI	
1760	1762	benzyl benzoate						MS-RI	
1774	1781	1-pentadecanol		1.14 ± 0.11				MS-RI	
1792	1792	1,2-15,16-diepoxy-hexadecane						MS	
1810	1816	*cis*-11-hexadecenal						MS-RI	
1827	1827	benzyl salicylate						MS-RI	[40]
1863	1864	1-hexadecanol	1.10 ± 0.11	1.14 ± 0.07	1.80 ± 0.15	1.37 ± 0.14		MS	
1863	1866	*cis*-9-hexadecen-1-ol	1.14 ± 0.09		0.19 ± 0.01		1.05 ± 0.09	MS-RI	
1890	1890	2-methylhexadecan-1-ol					1.05 ± 0.09	MS	
1946	1944	palmitic acid						MS-RI	
2046	2046	geranyl linalool						MS	
2052	2052	*cis*-*cis*-9,12-octadecadien-1-ol	17.73 ± 0.44	14.74 ± 0.36	9.72 ± 0.22	13.51 ± 0.39	15.37 ± 0.37	MS-RI	[22]
2058	2058	*cis-cis-cis*-9,12,15-octadecatrien-1-ol	4.59 ± 0.21	3.58 ± 0.27	7.53 ± 0.28	3.27 ± 0.12	2.98 ± 0.16	MS-RI	[41]
2060	2060	*cis*-9-octadecen-1-ol			4.51 ± 0.17			MS-RI	
2074	2074	*n*-octadecyl alcohol	0.92 ± 0.05	0.62 ± 0.04	0.71 ± 0.08			MS-RI	
2104	2104	12-methyl-*E*,*E*-2,13-octadecadien-1-ol						MS-RI	
2114	2114	phytol	0.94 ± 0.05	0.73 ± 0.04	1.46 ± 0.26	0.52 ± 0.02	1.46 ± 0.10	MS-RI	
2178	2178	linolenic acid						MS-RI	
2279	2279	methyl 11,14,17-icosatrienoate						MS-RI	
2241	2241	*trans-trans-cis*-1,3,12-nonadecatriene-5,14-diol						MS-RI	
		No. of identified constituents	37	40	42	40	27		
			96.75	94.84	95.19	96.65	93.41		

* ID = identification methods: MS by comparison of the mass spectrum with those of the computer mass libraries Adams and Nist 11, and by interpretation of the mass spectral fragmentations; RI by comparison of retention index with those reported in the literature; Std by comparison of the retention time and mass spectrum of available authentic standards; MS by identification of the mass spectrum. Nonpolar column ZB-5. Data are the mean of three replicates.

**Table 4:** Collecting stations for accessions of Sardinian and Spanish *B. bituminosa* (and its varieties) and endemic *B. morisiana* plants.

**Station (n°)**	**Coordinates**	**Soil Substrate**	**a.s.l.**	**Geomorphology**
**Collecting stations for Sardinian *B. bituminosa* var *bituminosa* plants**				
Loculi (1)	40°24′29.7″ N 09°36′25.0″ E	granite soil	26 m	flat land
Monte Rosello (2)	40°43′53.8″ N 08°33′32.2″ E	limestone	225 m	hill
Siniscola (3)	40°34′46.7″ N 09°41′38.4″ E	metamorphic rocks	38 m	flat land
**Collecting stations for Sardinian endemic species *B. morisiana* plants**				
Monte Gonareddu (4)	40°13′42.4″ N 09°11′53.1″ E	limestone	1035 m	mountain
Punta Giglio (5)	40°34′08.4″ N 08°12′16.7″ E	limestone	80 m	promontory
Siliqua (6)	39°15′07.7″ N 08°46′57.2″ E	limestone	87 m	flat land
Bitti (7)	40°28′26.0″ N 09°22′27.4″ E	Granit	740 m	mountain
Burcei (8)	39°20′56.8″ N 09°21′38.7″ E	schists	530 m	hill
**Collecting stations for Spain-Canary Islands *B. bituminosa* var. *albomarginata* plants**				
Caleta de Famara (9)	29°06′47.7″ N 13°33′20.5″ W	Fisures, gravel slopes	30 m	flat land
Arecife (10)	28°59′18.5″ N 13°32′07.1″ W	Road margins	54 m	flat land
**Collecting stations for Spain-Canary Islands for *B. bituminosa* var. *crassiuscula* plants**				
San Cristòbal de la laguna (11)	28°28′59.8″ N 16°18′00.0″ W	Rocks, gravel slopes	503 m	hill
Vilaflor (12)	28°09′32.5″ N 16°37′56.6″ W	Rocks, gravel slopes	1400 m	mountain
**Collecting stations for Spain-Canary Islands for *B. bituminosa* var. *bituminosa* plants**				
Puntas de Calnegre (13)	37°30′29.7″ N 01°24′09.2″ W	Siliceous, ruderal	25 m	flat land
Llano del Beal (14)	37°37′29.1″ N 00°50′29.6″ W	Nitrified road margins	105 m	flat land
	(1)	Rocks, gravel slopes	503 m	hill

**Table 5:** Yield of methanolic crude extracts of *Bituminaria* varieties.

*Bituminaria* Varieties (Station)	Yield: g/100 g; (Std. Error)
*Bituminaria bituminosa* var. *bituminosa* (Loculi—Sardegna)	1.70 (0.06)
*Bituminaria bituminosa* var. *bituminosa* (Monte Rosello (SS)—Sardegna)	1.60 (0.06)
*Bituminaria bituminosa* var. *bituminosa* (Siniscola—Sardegna)	2.00 (0.12)
*Bituminaria bituminosa* var. *bituminosa* (LIano del Beal—Spagna)	1.82 (0.09)
*Bituminaria bituminosa* var. *bituminosa* (Calnegre—Spagna)	2.40 (0.12)
*Bituminaria bituminosa* var. *bituminosa* (San Cristòbal de la Laguna—Tenerife)	2.20 (0.06)
*Bituminaria morisiana* (Monte Gonareddu-Sardegna)	2.18 (0.07)
*Bituminaria morisiana* (Punta Giglio—Sardegna)	1.90 (0.04)
*Bituminaria morisiana* (Siliqua—Sardegna)	1.98 (0.07)
*Bituminaria morisiana* (Burcei—Sardegna)	1.52 (0.10)
*Bituminaria morisiana* (Bitti—Sardegna)	1.78 (0.12)
*Bituminaria bituminosa* var. *albomarginata* (Arecife—Lanzarotte)	2.00 (0.12)
*Bituminaria bituminosa* var. *albomarginata* (Caleta de Famara -Lanzarotte)	1.91 (0.04)
*Bituminaria bituminosa* var. *crassiuscula* (San Cristòbal de la Laguna—Tenerife)	2.08 (0.05)
*Bituminaria bituminosa* var. *crassiuscula* (Vilaflor—Tenerife)	1.90 (0.03)

## Sample Availability

Samples of the compounds are not available from the authors.
